# Fourth UNHLM on noncommunicable diseases 2025: An opportunity to bridge the transcending priorities for impact in global south

**DOI:** 10.1371/journal.pgph.0004287

**Published:** 2025-03-19

**Authors:** Cherian Varghese, Anirudh Prem, Baridalyne Nongkynrih, Vijay Kumar Chattu, Bente Mikkelsen

**Affiliations:** 1 Prasanna School of Public Health, Manipal Academy of Higher Education, Manipal, Karnataka, India; 2 Centre for Community Medicine, All India Institute of Medical Sciences, New Delhi, India; 3 Department of Community Medicine, Faculty of Medicine, Datta Meghe Institute of Medical Sciences, Sawangi, India; 4 Department of Occupational Science and Occupational Therapy, Temerty Faculty of Medicine, University of Toronto, Toronto, Ontario, Canada; 5 Independent Advisor, Oslo, Norway; PLOS: Public Library of Science, UNITED STATES OF AMERICA

## A journey of commitments at the highest level

Non-communicable diseases (NCD) remain a critical global health challenge accounting for 74% of deaths and 86% of premature deaths due to NCDs in low- and middle-income countries (LMICs) in 2023 [1]. Most countries are not on track to achieve the SDG 3.4 target of reducing premature mortality by one-third by 2030.

The first Political Declaration on NCDs in 2011 included strong commitments for prevention and control of NCDs. The 2013 World Health Assembly adopted a comprehensive global monitoring framework (GMF) and a Global Action Plan on NCDs [2], while the second UNHLM in 2014 reaffirmed the commitments [3]. The 2018 Political Declaration (PD) introduced time-bound national commitments, greater accountability and increased financing for NCDs [4]. It recognised the significance of mental health, commercial determinants of health, and emphasis on UHC alongside the importance of private sector and civil society [5].The upcoming 4th UNHLM on NCDs provides a critical opportunity to realign priorities and commitments along with learnings from the COVID-19 pandemic.

## Progress (or the lack of) in NCD prevention and control

Despite global advocacy and efforts, the commitments remain insufficient, and progress remains slow and unevenly distributed. The 2022 WHO NCD progress monitor indicates that 126 out of 194 countries have set national targets and 77 countries achieved an improvement on NCD control [6]. However, these have not translated to the reduction in premature deaths required to meet the SDG 3.4 target. The fact that no Member State in the WHO African Region has completely fulfilled the NCD monitoring indicators highlights the inequalities in the implementation of NCD control. The increasing trends obesity [7] and diabetes from 1990 to 2022 across the globe [8] are a stark reminder that attempts so far have not made any dent.

An area of progress is in tobacco control, with more than half of all countries fully achieving the implementation of plain/standardized packaging and/or large graphic health warnings on tobacco packaging. However, risk factor reduction interventions such as alcohol, sugar and salt, remain inadequate [9].

The fastest declines in premature mortality were observed in European and Western Pacific regions. Premature deaths in the Eastern Mediterranean Region remained high, but the Southeast Asia Region showed stagnation in 2000–2019. The Region of the Americas stayed at the lowest level, while the African Region saw a more moderate decline. Mortality by world income group shows a declining trend, and as expected, premature mortality is lowest in high-income countries as compared to low-income countries [10].

## Insights and inputs for the 4th UNHLM on NCDs

Health system capacity is inadequate in LMICs across all six building blocks of the health system [11], and the non-binding nature of mechanisms in Political Declarations has undermined the accountability of national action for NCD control and for global health security. There is no escape but to invest in capacity of health systems to meet the huge unmet needs for NCD programs in LMICs. The Lusaka Agenda on the future of Global Health Initiatives calls on these institutions and their holders to ensure their role as an entry point and catalyst for accelerating change in the global health ecosystem. The shifts proposed in the call are a good way to align global support for NCDs [12]. Efforts in hypertension control have shown that PHCs can be effective in managing uncomplicated NCDs and can function as ‘gate keepers’ to optimize referral care [13]. Interventions such as HPV single-dose vaccinations, screening and treatment should be a priority in LMICs and should become targets in the 2025 UNHLM [14]. The next UNHLM should have explicit targets reflecting the coverage of hypertension management and HPV vaccination coverage.

**Fig 1:**
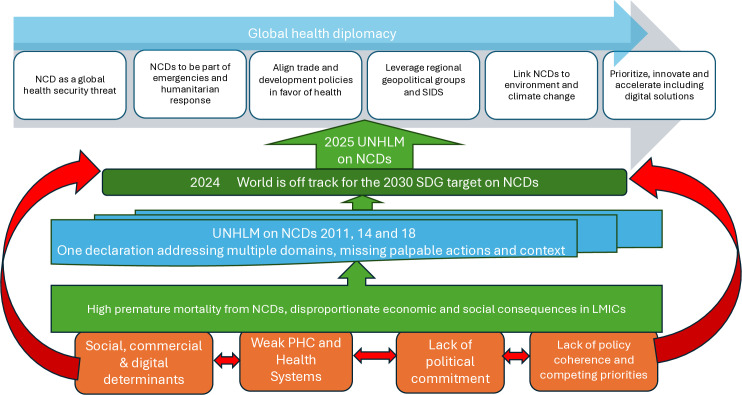
Framework of current status and priority focus areas for UNHLM on NCD 2025 (*Source: prepared by the authors*).

To achieve 2030 SDG targets, there needs to be a change of perspective and priorities to better reflect the epidemiological and economic realities of LMICs and ensure equitable progress toward global NCD targets. Fig 1 summarizes the global challenges and a pathway towards more equitable and realistic progress.

With the upcoming 4th United Nations High Level Meeting on NCDs expected in September 2025, we offer the following recommendations to truly make progress in combatting NCDs around the world:

- The 4th UNHLM PD should present priorities and commitments by groups of countries with varying levels of income and promote allocation of global resources for those who are most in need. Groups such as the Small Island Developing States, lower income countries and economies in transition with a double burden of disease will need targeted attention in the PD. The PD should guide the operationalization of social, economic, commercial, and environmental influences on NCDs and should consist of bold statements from Heads of State or Government for policy coherence keeping health at the centre.
- The PD should propose actions to address the disproportionate funding for NCDs, as global funding for health is still predominantly on communicable diseases. Low- and middle-income countries must implement NCD interventions and funding should be earmarked for these countries. Health systems in these countries also need substantial support to make them NCD ready and to achieve Universal Health Coverage.
- The need for a decentralized and differentiated approach to implement the commitments should be explicit. The PD should have recommendations to groups such as SIDS and LMICS separately and cannot leave it to a generic guidance to adapt countries in their national context.
- The PD should mandate accountability using tracers at global level such as the proportion of financing for NCDs, catalytic support for NCDs in LMICS and SIDS, and at the national level a small set such as HPV vaccination coverage, tobacco prevalence and hypertension control.

The world must address the barriers to effective prevention and NCD control, particularly in the Global South, and the opportunity at the 4th UNHLM should be maximized. We propose targeted, flexible, and interdisciplinary approaches that consider the context of LMICs along with bold and accountable actions from the Heads of States and Governments.
